# The Use of Antifreeze Proteins in the Cryopreservation of Gametes and Embryos

**DOI:** 10.3390/biom9050181

**Published:** 2019-05-09

**Authors:** Vanesa Robles, David G. Valcarce, Marta F. Riesco

**Affiliations:** 1 Spanish Institute of Oceanography (IEO), 39012 Santander, Spain; dgvalcarce@gmail.com (D.G.V.); riesco.mf@gmail.com (M.F.R.); 2 MODCELL GROUP, Department of Molecular Biology, Universidad de León, 24071 León, Spain

**Keywords:** AFP, cryopreservation, vitrification, embryo, spermatozoa, oocytes

## Abstract

The cryopreservation of gametes and embryos is a technique widely used in reproductive biology. This technology helps in the reproductive management of domesticated animals, and it is an important tool for gene banking and for human-assisted reproductive technologies. Antifreeze proteins are naturally present in several organisms exposed to subzero temperatures. The ability for these proteins to inhibit ice recrystallization together with their ability to interact with biological membranes makes them interesting molecules to be used in cryopreservation protocols. This mini-review provides a general overview about the use of antifreeze proteins to improve the short and long term storage of gametes and embryos.

## 1. Introduction

**Figure 1:**
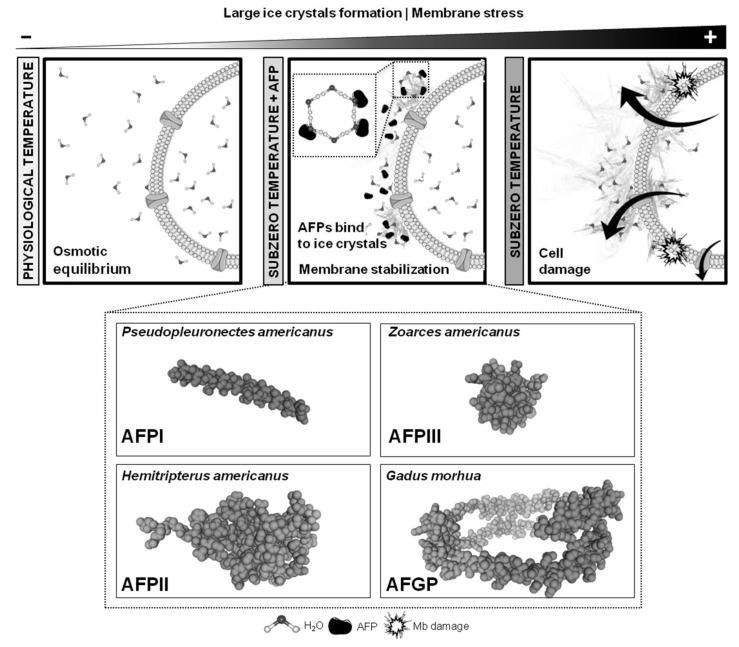
Antifreeze protein (AFP) mechanisms of the action binding to forming ice crystals around gametes or the embryo, and 3D models of the most used AFPs in reproductive technologies. The 3D models were created using the SWISS-MODEL online platform with the following National Center for Biotechnology Information (NCBI) sequences: AFPI (*Pseudopleuronectes americanus*; GeneBank ID: AAA49466.1), AFPII (*Hemitripterus americanus,* GenBank ID: AAA49618.1), AFPIII (*Zoarces americanus;* GenBank ID: ABA41371.1), and AFGP (*Gadus morhua*; GenBank ID: AAQ09567.1)

There are several species that naturally display a special protection against low temperatures thanks to their ability to produce an intriguing group of proteins that binds to ice, preventing ice crystal growth. These particular proteins, called antifreeze proteins (AFPs), could be present in bacteria, fungi, crustacean, microalgae, insects and fish (AFPs type I, II and II) [1,2,3,4,5,6,7,8,9,10,11,12,13] (Figure 1). In the natural environment, these proteins are crucial for animal survival at freezing temperatures. Thermal hysteresis and ice recrystallization inhibition, together with their ability to interact with biological membranes, are important properties of AFPs (Figure 1). It is interesting to notice that different AFPs could have a different activity and that they bind to different faces of the ice crystal [14]. Their ability to inhibit ice recrystallization is crucial to protecting membranes against freezing injuries [5,14,15,16,17,18,19]. This last property of AFPs, together with their capacity to interact with cellular membranes, makes these proteins interesting molecules for being incorporated in cryopreservation protocols. However, this property is dependent of the type of AFP and the type of membrane, i.e., the bilayer lipid composition of the membrane determines the interaction way with a specific AFP, as well as their protection ability [19].

Cryopreservation is a widely used technique in reproductive biotechnology. In humans, it is an important tool for assisted reproductive technologies (ARTs). Both embryo and gametes cryopreservation are routine procedures in clinical practice [20,21]. However, cryopreservation is also very relevant for the reproductive management of domesticated animals, including fish. In this last group of animals, only sperm cryopreservation has been successfully achieved, given that oocyte and embryo cryopreservation are very challenging due to their big size, the great amount of vitelum, and in the case of embryos, their multi-compartmental structure [22,23].

The use of cryoprotective agents is a crucial aspect in all cryopreservation protocols, from slow freezing to vitrification. There are non-permeable (or external) and permeable (internal) cryoprotectants, depending on their ability to permeate biological membranes. Some examples of non-permeable cryoprotectants are glucose, sucrose or polyvinylpyrrolidone (PVP). On the other hand, some permeable cryoprotectants are dimethyl sulfoxide (DMSO), 1,2-propanediol, glycerol, ethylene glycol and methanol, among others [24]. Considering the interesting cryoprotective properties of AFPs, some researchers have tried to include these proteins in their designed cryopreservation protocols, either in the extender or by injecting them in specific compartments such as the yolk of fish embryos (addressed in the Section 2 and Section 3 of this review). It is also interesting to mention that some of the commonly used cryoprotectants have a high toxicity. Thus, the possibility of using AFPs in such protocols, reducing the amount of toxic cryoprotectants, is also an interesting aim, providing a potential improvement on the efficiency and safety of cryopreservation protocols. This review provides a general and updated overview of the different approaches to using AFPs in gamete and embryo cryopreservation, from fish to mammals (Table 1).

## 2. Use of Antifreeze Proteins in Sperm Cryopreservation

The cryobanking of reproductive cells is a useful tool that provides benefits for animal husbandry programs, human infertility treatments and biomedical research [25,26,27]. Spermatozoa are the most frequent resource in cryobanking due to their characteristics that simplify sample collection and preservation when compared to embryos and oocytes. However, sperm preservation protocols vary among species due to their inherent characteristics (size, morphology, membrane and acrosome composition). In fact, spermatozoa from different species could have different tolerances to cryodamage. Several factors during the freezing process, including sudden temperature changes, ice formation and osmotic stress, have been proposed as the main causes for poor sperm quality after thawing [27]. Despite the extensive progress that has been reached in this field, new treatments that could overcome the mentioned bottlenecks are necessary. In this respect, AFP supplementation could represent a promising alternative.

The first studies on AFP supplementation to improve spermatozoa storage were focused on testing different types of AFPs at different concentrations (0.1 to 10 µg/mL) in the extender. In ram, AFP type I and antifreeze glycoproteins (AFGP) type I enhanced post-thaw motility when used at 10 µg/mL concentration [28]. Recently, the positive effect of AFPs on sperm motility after thawing, specifically of AFPIII at 1 µg/mL, has been demonstrated in other species such as the Japanese white rabbit [29]. In the case of buffalo, different articles have been published trying to improve sperm freezability by AFP addition in the cryoprotectant medium [30,31,32]. A variety of AFPs, including the *Dendroides canadensis* recombinant antifreeze protein (DAFP), were tested in different conditions, reporting different beneficial effects in cold storage (4 °C) and during freezing. After freezing/thawing, authors reported an increase in progressive motility and plasma membrane integrity using 0.1 µg/mL AFPIII, finding that higher concentrations were inefficient [30]. In another work, the role of Antarctic fish AFGPs was evaluated by the same group in this species. A rise in motility and a better plasma membrane integrity were registered after thawing using 1 µg/mL AFGP [31]. Finally, a recent study performed by the same group demonstrated the positive effects on sperm motility and membrane integrity after a 10 µg/mL AFP supplementation [32]. In these studies, the authors did not observe any significant outcome derived from the AFP supplementation when sperm samples were cooled at 4 °C instead of being cryopreserved. The effect of the AFP presence in the extender during the chilling and freezing procedures has been also studied in other species [28,33]. In the case of chimpanzee spermatozoa, a significant improvement on motility, plasma membrane and acrosome integrity was registered after cryopreservation with AFP supplementation [33]. However, a mild cytotoxic effect was observed in all AFP concentrations tested after chilling (0–5 °C) [33]. This result was supported by a previous study performed in ram, in which AFP and AFGP, at 0.1 and 100 pg/mL concentrations, were mildly cytotoxic to spermatozoa cooled at 5 °C [28]. This fact was explained by Carpenter and colleagues, who described a balance between the AFP-induced enhancement of cell preservation and AFP-induced enhancement of cell damage [34]. In this balance, the inhibition of ice recrystallization and the preferential growth of ice around the cells are crucial factors [34].

Sperm survival and motility are not the only parameters susceptible to improvement after cryopreservation [35]. In a study performed in bovine sperm, a supplementation with AFPI, AFPIII and AFPG, at four concentrations, did not improve sperm viability. However, an increase in osmotic resistance was registered in cells which were exposed to AFPI at 0.1, 1 and 10 mg/mL before cryopreservation [36]. In this work, the authors suggested that the beneficial effect in terms of the osmotic resistance was due to a decrease in the membrane mechanical stress [36]. As we have reviewed, the beneficial effects of antifreeze proteins in the male gamete cryopreservation have been widely demonstrated in mammals; however, some exceptions have been observed. The AFP concentration must be carefully optimized in order to avoid negative effects on cell survival. In mouse spermatozoa cryopreservation, the addition of AFPI, AFPIII and antifreeze glycoprotein at concentrations of 0.01–100 µg /mL in the extender did not maintain the sperm quality after thawing [37]. The authors argued that the drop in survival might be related to physical or mechanical damage after the freezing and thawing processes. This deleterious effect, associated to mechanical damage mediated by AFPs, has been previously observed and discussed by other authors [34,38] in red blood cell cryopreservation. In these studies, authors described an alteration of the ice crystal shape provoked by AFP supplementation at high concentrations and their detrimental effect on cell survival. Specifically, AFP addition in the mg/mL concentration range alters the crystal morphology from dendritic to spicular or needle shaped ice crystals, resulting in a decrease of survival triggered by this new acquired ice conformation [39]. The same detrimental effect was observed by our group in zebrafish primordial germ cell (PGC) cryopreservation using 10–20 mg/mL AFPI [40].

Antifreeze proteins have been also used to improve fish sperm chilling resistance and freezability. The first studies performed in carp sperm were focused on decreasing the cryoprotectant concentration, avoiding possible toxic consequences during hypothermic conditions [41]. In this protocol, different AFGPs were added to the extender medium during storage at 4 °C, registering a significant decrease in sperm mortality [41]. After these preliminary analyses, the AFP supplementation was evaluated in gilthead seabream. This species is considered an interesting model to study sperm cryodamage, taking into account that there is still a margin of optimization for this purpose in this fish [42,43,44,45]. The effect of AFP-supplemented cryopreservation on lipid profiles in seabream has been explored. The phospholipids and fatty acid composition of spermatozoa suffered an alteration as a consequence of the AFP treatment. This alteration indicated a direct effect of the AFPs on the fatty acid stabilization in the membrane, preventing the sublethal damage caused by the sperm membrane disturbance during the cryopreservation procedures [46]. Moreover, the authors also observed a higher percentage of viable cells and a higher values for the VSL (straight line velocity) and LIN (linearity) parameters after 1 µg/mL of AFPIII incubation and cryopreservation [46].

All the previous studies analyze the effect of AFPs on sperm survival, functionality and plasma membrane composition. However, AFP supplementation could also avoid other molecular alterations. Zilli and colleagues demonstrated that an AFPIII addition improves seabream spermatozoa viability, motility and straight-line velocity, but they go further in detail, analyzing the protein profile in experimental groups [47]. They concluded that the alteration in the seabream spermatozoa protein profile after cryopreservation is significantly reduced in those samples that were frozen with AFPIII. Specifically, the authors detected a decreased level of some proteins related to the bioenergetic system of the cell, such as glyceraldehyde 3-phosphate dehydrogenase (GAPDH) and malate dehydrogenase thermolabile form (MDH) [47].

Recent studies conducted in other fish species, such as sturgeon, claimed that 10 µg/mL AFPIII is effective in improving sperm motility and in the preserving spermatozoa energetic metabolism after cryopreservation [48]. It is also interesting to notice that AFPIII supplementation provides higher motility rates after carp sperm cryopreservation than purified seminal plasma transferrin (a compound with antioxidant properties). This effect was probably observed due to AFPIII’s higher capacity of binding to the cell membrane and of therefore promoting a higher membrane stabilization than transferrin [49].

## 3. Use of Antifreeze Proteins in Oocyte Cryopreservation

Nowadays, oocyte cryopreservation in some mammal species is an established technology with a wide range of applications. This technology has registered tremendous progress and development in the last years, and germline cryobanking in this cell type is a valuable tool. They are better candidates than embryos for cryopreservation, e.g., they are smaller in size, have a higher membrane permeability, are less sensitive to chilling and have a less complex membrane system, which affect their survival and developmental competence after cryopreservation [23]. However, the cryopreservation of oocytes is still a challenging task in some species, such as fish species or bovine oocytes, due to the lack of protocols or due to an inefficient cryopreservation protocol, which is why optimization is imperative [50]. In this scenario, different attempts have been performed to improve the current cryopreservation protocols, including AFP supplementation.

A study using different types of AFPs at different concentrations to improve oocyte vitrification was performed in mice and pigs. In both species, AFPs increased the survival (82% and 25% respectively) [51]. Moreover, in this study the authors concluded that the protective mechanism of AFP is unrelated to the inhibition of ice crystal growth. The authors proposed that the main action mechanism of these proteins is related to cell membrane stabilization. In addition, the dose response curves of the protective effect showed saturation kinetics of 20 mg/mL [51]. These findings were confirmed in pig oocyte cryopreservation using different AFP types (AFPI, II, III and AFGP) and by evaluating the morphology and oolema integrity through Fluorescein Diacetate (FDA) staining [52]. In this work, the authors supported previous results that claimed that AFP proteins had a similar ability to interact with and protect the oolema [52]. After these studies, other authors made an effort to optimize the AFP supplementation conditions, through, for example, the different stepwise addition of cryoprotectants at room temperature (19–21 °C) or on ice (2–4 °C) to decrease the variability in the results. In these studies on mouse oocytes, the low temperatures in the cryoprotectant exposure steps improved the viability after the vitrification with 6 M dimethyl sulfoxide supplemented with 1 mg/mL AFGP. Moreover, the authors remarked that the AFGP supplementation at room temperature removed its positive effect on the oocyte viability [53].

New molecular quality analyses were employed to define the beneficial effect of 500 ng/mL AFP supplementation during vitrification procedures in mouse oocytes [54,55]. In these assays, a set of genes related to the spindle checkpoint was analyzed, taking into account its role in chromosome segregation. Moreover, other parameters, such as the ATP content, demonstrated the positive effects of AFP addition in mouse oocytes prior to cryopreservation [54,55]. Other integrative studies have been published comparing the effect of new AFP types (*Flavobacterium frigoris IBP*, FfIBP, *Glaciozyma sp*. IBP, formerly known as *Leucosporidium sp*., LeIBP and Type III AFP) in the same oocyte cryopreservation protocol [3,8]. In these studies, the post-thawed analyses were performed in terms of survival, development rates and the in vitro fertilization ability after vitrification [56]. The authors concluded that among the three AFPs, FfIBP was the most effective for protecting against cryodamage during vitrification, preserving the oocyte structure and promoting ice growth inhibition.

Recently, the effectiveness of AFPG III and 8 supplementation during bovine oocyte vitrification has been demonstrated [50,57]. The AFP positive effects were related to several parameters, such as: the damage reduction of oocyte actin filaments, the preservation of the spindle and chromosome dynamics and the mitochondrial distribution. 

## 4. Use of Antifreeze Proteins in Embryo Cryopreservation

The first reports on embryo cryopreservation date back to 1972, when Whittingham and Wilmut successfully achieved an 8-cell mouse embryo cryopreservation [58]. Later, in 1983, Trounson and Mohr achieved a human pregnancy from a frozen embryo [59]. Embryo cryopreservation may not only be considered to be a method for embryo storage. Some authors defend the "Theory about the Embryo-Cryo treatment", arguing that freezing and thawing could activate repair mechanisms in preimplantation embryos [60]. In humans, embryo cryopreservation provides benefits at different levels: it gives the opportunity for genetic testing, provides ART patients with additional embryo transfers (increasing the changes of the pregnancy from a single stimulated cycle) and helps patients suffering from cancer to store embryos prior to chemotherapy [61]. In animals, embryo cryopreservation would have clear benefits in reproductive management and for gene banking purposes.

Cryopreservation can produce important injuries in embryos because the cold and osmotic shock, ice formation, or cryoprotectant toxicity could all damage the cell structure and function. Therefore, the design of the cryopreservation protocol is a crucial step for guaranteeing embryo survival after freezing/thawing. The choice of optimal freezing/thawing rates and the selection of the most adequate combination of cryoprotectants (CPAs) are very important aspects that will determine the success of the procedure. Usually, when a combination of cryoprotectants must be used, and particularly when a high concentration of cryoprotectants must be employed (for example in vitrification), a stepwise manner of CPA exposure prior to freezing could be recommended. In this way, the CPA concentration is gradually increased, and the embryos are only exposed to the highest CPA concentration for a short period of time at the end of the CPA incorporation protocol. Furthermore, in some species, the last steps of the CPA incorporation protocol prior to cryopreservation can be done at low temperatures (e.g., 4 °C) in order to decrease the toxicity [62]. Usually, permeable CPAs are present from very early on, whereas non-permeable CPAs are included only in later stages.

Several authors have considered that AFPs could be interesting molecules to be included in cryopreservation protocols to protect cells from injuries, mainly due to their ability to interact with cell membranes and their capacity to inhibit ice crystal growth. Several studies on the short or long term storage of embryos, performed with bovine, ovine, equine and porcine embryos as well as with embryos from rabbits, mice and different fish species (zebrafish, turbot, seabream), use protocols that include these proteins in their design [19]. Interestingly, all of the assays performed in embryo cryopreservation used AFPs having a fish origin (mainly AFPI, AFPIII and AFGP) (Figure 1). Given that these proteins are naturally expressed in some arctic and/or subarctic species, to perform cryopreservation assays directly using embryos from such species could be an interesting approach. Robles et al. [62] vitrified embryos from the Winter flounder (*Pseudopleuronectes americanus*), a subarctic species that produces type I AFP and a hyperactive antifreeze protein comparable to those produced by insects [63]. In this study, two vitrification protocols with a step wise CPA incorporation protocol were used. With one of them (5M Me_2_SO, 1M EG, 2M Methanol and 10% sucrose), the authors reported embryo survival after thawing; however, low survival rates were achieved and none of the embryos were able to hatch [62]. Apart from this study, in fish, AFPs have been used by being added in the media [64] and microinjected in the yolk of the embryo [65,66]. In a study carried out by Robles et al. (2006), the authors confirmed that Fluorescein isothiocyanate (FITC)-labeled AFPIII can be successfully microinjected and homogeneously distributed in the yolk of turbot embryos. The same authors reported in 2007 [66] that AFPI (10 mg/mL) significantly increases the chilling resistance at 0 °C of *Sparus aurata* embryos microinjected in the yolk with 20 nl of the protein. When present in the extender, AFPs could improve cell survival after the cryopreservation of zebrafish dissociated the blastomeres [67]. In this last article, the authors improved cell survival from 29.9% (obtained using 2M DMSO as CPA) to 54.3% when AFP I was present in the cryoprotectant solution.

To summarize, in fish, AFPs could be useful for improving the embryo hypothermic storage or improving the viability of dissociated blastomeres after cryopreservation. However, their addition, either in the extender or microinjected in the yolk, is not sufficient to overcome the major underlying problems in fish embryo cryopreservation.

In mammals, we also have a complex scenario. In bovine embryos, the use of AFPIII improved the embryo development on day 5 of culture, although this effect was not maintained up to the blastocyst formation [50]. Other work reported improvements in bovine embryos’ short term storage (4 °C), combining the addition of AFP in the media (10 mg/mL) with a controlled prewarming [68]. Moreover, supplementation with AFGP8 during vitrification seems to have a protective effect against chilling-induced injury in bovine blastocysts [69]. In rabbits, 500 ng/mL AFPIII increased the embryo survival after an ultra-rapid vitrification, but 1000 ng/mL decreased it. This result points to an important factor that has been previously mentioned and must be taken into account when using these proteins, which is the fact that the protein concentration could determine the success of the procedure [29]. Mouse embryos cryopreserved in the presence of AFPs had a better morphological preservation (87.5%) than those embryos that were only cryopreserved with traditional cryoprotectants: with 1 M and 3 M DMSO (53.8 and 71.1%, respectively). However, they were non-viable and did not develop in culture [70]. Another study, carried out in mouse pronuclear and 4-cell embryos that were cryopreserved, concluded that adding antifreeze proteins of type I or III did not improve the survival or development [71]. In equine embryos, no improvement in the cryoprotection was obtained when using AFP in the cooling or freezing solutions [72], although it must be noted that a very low number of embryos were used in the study. Finally, in porcine embryos the supplementation of AFPIII (1 µg/mL) did not have beneficial effects on the development of embryos stored at hypothermic temperatures [73].

## 5. Conclusions

In conclusion, the benefits of the use of AFP to improve gametes’ and embryos’ survival after cold storage and cryopreservation are quite controversial and variable. In sperm, some studies reported improvements, mainly in terms of motility and viability. In oocytes and embryos, improvements in terms of survival have been reported after short or long-term storage. However, in most cases, the results, although promising, are quite variable, and success seems to depend on the species, cell type or embryo developmental stage, AFP type, AFP concentration and also on the cryopreservation protocol that is used. We can come to the conclusion that AFPs could provide some benefits when used at the optimal concentration but only when used in combination with an adequate and well-designed cryopreservation protocol. Further studies could clarify the possibility of using a combination of AFPs from different origins: insects [32], fish [64] or bacteria [56,74], to explore if synergic effects are reported. Protein engineering that includes different domains is another option that may provide new opportunities to improve the AFP use for the optimization of cryopreservation protocols. Moreover, the possibility of directly delivering the AFPs within the cytoplasm could be an interesting approach for controlling ice crystal growth within the cell. Femtosecond laser technology successfully delivers molecules (proteins and DNA molecules) within the cell, avoiding cell damages [75], and this could be an interesting technique to use for these purposes.

## Figures and Tables

**Table 1:** Main studies using antifreeze proteins (AFPs) in the cryopreservation of gametes and embryos.

AFPs	Organisms	Sample Type	Concentration	Freezing Method	Year
AFPI	Zebrafish	Primordial Germ Cells	10 mg/mL	Vitrification/ Cryopreservation	2011
AFPI-AFGP	Ram	Spermatozoa	0.1 µg/mL-10 µg/mL	Chilling/cryopreservation	1994
AFPIII	Chimpanzee		1, 10 and 100 µg/mL	Chilling/cryopreservation	1998
AFPI-AFP III-AFGP	Mouse		1-100 µg/mL	Cryopreservation	2002
AFPI-AFP III-AFGP	Bull		0.1, 1, 10 and 100 µg/mL	Cryopreservation	2006
AFPIII	Rabbit		0.1, 1, 10 and 100 µg/mL	Cryopreservation	2014
AFPIII	Buffalo		0.1, 1 and 10 µg/mL	Chilling/cryopreservation	2014
AFGP	Buffalo		0.1, 1 and 10 µg/mL	Chilling/cryopreservation	2015
DAFPs	Buffalo		0.1, 1 and 10 µg/mL	Cryopreservation	2016
AFGP	Common carp		2 and 10 mg/mL	Hypothermic	2002
AFPI-AFPIII	Gilthead seabream		1 µg/mL	Cryopreservation	2011
AFPI-AFPIII	Gilthead seabream		1 µg/mL	Cryopreservation	2014
AFPIII	Persian sturgeon		5, 10 and 15 µg/mL	Vitrification	2017
AFPI-AFPIII	Common carp		0.1, 1, 10 and 100 µg/mL	Cryopreservation	2019
AFGPs	Mouse/pigs	Oocytes	20 mg/mL	Vitrification	1992
AFPI-AFP II-AFPIII-AFGP	Pig		20 mg/mL	Vitrification	1993
AFGP	Mouse		1 mg/mL	Vitrification	1998
AFP	Mouse		500 ng/mL	Cryopreservation	2011
AFPIII	Mouse		2.5 mg/mL	Vitrification	2014
FflBP-LeIBP-AFP III	Mouse		0.1, 0.05 and 0,1 mg/mL (respectively)	Vitrification	2015
AFGPIII-8	Bovine		500 and 1000 ng/mL	Vitrification	2016
AFGP	Mouse	Embryos	20 mg/mL	Cryopreservation	1994
AFPIII	Mouse		0.1, 1 mg/mL	Cryopreservation	1995
AFP	Equine		20 mg/mL	Chilling/cryopreservation	1997
AFPIII	Rabbit		500 ng/mL	Cryopreservation	2014
AFP11	Bovine		10 mg/mL	Hypothermic	2015
AFPIII	Bovine		500 and 1000 ng/mL	Vitrification	2016
AFGP8	Bovine		1 mM	Vitrification	2017
AFPIII	Pig		1 µg/mL	Hypothermic	2018
AFPIII	Turbot		10 mg/mL	Hypothermic	2006
AFPI	Seabream		10 mg/mL	Vitrification	2007
AFPI-AFPIII	Zebrafish		40 µg/mL	Hypothermic	2008
AFPI-AFPIII	Zebrafish		40 µg/mL	Cryopreservation	2009
